# The combination of sorafenib and radiation preferentially inhibits breast cancer stem cells by suppressing HIF-1α expression

**DOI:** 10.3892/or.2013.2228

**Published:** 2013-01-08

**Authors:** JAE HO LEE, JAE WOONG SHIM, YOO JIN CHOI, KYU HEO, KWANGMO YANG

**Affiliations:** 1 Research Center, Dongnam Institute of Radiological and Medical Sciences, Busan, Republic of Korea; 2 Biospectrum Life Science Institute, Seongnam-si, Gyeonggi-do, Republic of Korea; 3 Department of Radiation Oncology, Dongnam Institute of Radiological and Medical Sciences, Busan, Republic of Korea; 4 Department of Radiation Oncology, Korea Institute of Radiological and Medical Sciences, Seoul, Republic of Korea

**Keywords:** breast cancer stem cell, sorafenib, radiation, hypoxia

## Abstract

The importance of anticancer stem cell research for breast cancer lies in the possibility of providing new approaches for an improved understanding of anticancer activity and cancer treatment. In this study, we demonstrated that the preclinical therapeutic efficacy of combining the multikinase inhibitor sorafenib with radiation was more effective in hypoxia-exposed breast cancer stem cells. We assessed cell viability and Annexin V to evaluate the combined effect of sorafenib and radiation following exposure to hypoxia. Our results showed that the synergistic cytotoxicity increased tumor cell apoptosis significantly and reduced cell proliferation in MDA-MB-231 and MCF-7 cells under hypoxic conditions compared to sorafenib or radiation alone *in vitro*. Additionally, the combined treatment induced G2/M cell cycle arrest. Notably, the combination of sorafenib and radiation eliminated CD44^+^CD24^−/low^ cells preferentially, which highly expressed hypoxia-inducible factor (HIF)-1α and effectively inhibited primary and secondary mammosphere formation in MDA-MB-231 cells. A combined effect on MDA-MB-231 cells in response to hypoxia was shown by inhibiting angiogenesis and metastasis by suppression of HIF-1α and matrix metalloproteinase-2 (MMP-2). Collectively, these results indicate that the efficacy of sorafenib combined with radiation for treating human breast cancer cells is synergistic and suggest a new therapeutic approach to prevent breast cancer progression by eliminating breast cancer stem cells.

## Introduction

Breast cancer is the second most common cancer among Korean women. Numerous studies have been conducted on treatments for patients with breast cancer, as most treatments with radiotherapy and chemotherapy fail, and recurrence and metastasis often occur. Therefore, it is crucial to discover new therapies than can reduce breast cancer mortality. General conventional therapy does not kill all tumor cells and a small side population of cells, which are resistant to radiation and drugs, might be the origin of recurrence and metastasis. These cells are referred to as cancer stem cells (CSCs) (1), and are tumor initiating cells that propagate tumors phenotypically similar to the parental tumor.

Breast CSCs have been isolated by sorting CD44^+^CD24^−/low^ cells to initiate the process of carcinogenesis in NOD/SCID mice (2). Tumor growth, invasion, and metastasis depend on the properties of CSCs and their interactions with the tumor microenvironment (3). The importance of the tumor niche has been highly recognized. Among numerous factors to explore tumor microenvironments known to associate with aggressive CSCs (4), hypoxia is believed to be crucial and promotes aggressive tumor phenotypes (5). Oxygen tension is an important signal to induce the low-oxygen CSC population associated with maintaining undifferentiated cells (6). The expression levels of hypoxia-related proteins, including hypoxia-inducible factor-1α (HIF-1α), mediate the increase in the number of CSCs under hypoxic conditions (7). Theoretically, CSCs can be induced by anti-angiogenic therapies under hypoxia, conferring radioresistance to the CSCs, although this has yet to be demonstrated *in vivo*, and its clinical significance remains unknown.

Therefore, hypoxia-induced stimulation of CSCs might limit the effectiveness of radiotherapy and chemotherapy. These studies suggest that radiotherapy and chemotherapy might have to be combined with cancer stem cell-targeting strategies to improve patient recovery (7).

Sorafenib is an oral multikinase inhibitor (8,9) that blocks tumor cell proliferation and angiogenesis by inhibiting a Raf serine/threonine kinase and vascular endothelial growth factor (VEGF) receptors (10). Sorafenib is currently being used in clinics to treat patients with advanced renal cell carcinoma (RCC) and hepatocellular carcinoma (HCC) (11,12). Eliminating the CD44^+^CD24^−/low^ cell population from breast cancer cells occurs by inhibiting RAF-β-catenin activation *in vitro*(13). In addition, sorafenib effectively reduces melanoma, breast, colon, and lung cancer *in vivo*(10,14).

Sorafenib increases HIF-1α expression in melanoma cells (15); however, the anticancer mechanism of sorafenib combined with ionizing radiation has yet to be investigated. In the present study, we examined the efficacy of sorafenib and radiation against breast cancer and CSCs in non-metastatic MCF-7 and metastatic MDA-MB-231 cells. We demonstrated that a combination of sorafenib and radiation more effectively eliminates CSCs, which might be reflected by the inhibition of HIF-1α expression from metastatic MDA-MB-231 cells rather than non-metastatic MCF-7 cells under hypoxic conditions. Collectively, our results demonstrated that sorafenib and radiation can be successfully combined to potentiate anti-CSC and anti-angiogenesis activities.

## Materials and methods

### Cell culture

The human breast cancer cell lines MDA-MB-231 and MCF-7 were obtained from the American Type Culture Collection (Rockville, MD, USA) and maintained in DMEM (Welgene, Daegu, Korea) supplemented with sodium pyruvate (1 mM), 10% fetal bovine serum (FBS; HyClone, Logan, UT, USA), and 2% penicillin/streptomycin (Gibco, Carlsbad, CA, USA). Cells were cultured at 37°C in a humidified atmosphere of 5% CO_2_. Cells were maintained under hypoxic conditions in a glove box-type anaerobic chamber (Thermo Forma, Marietta, OH, USA). The hypoxic atmosphere was <1% O_2_, 5% CO_2_, 10% H_2_, and 85% N_2_ with continuous computerized monitoring, indicating a partial O_2_ pressure of <15 mm Hg at 37°C. O_2_-dependent experiments were performed in both hypoxic and normoxic incubators. Cells were irradiated with 10 Gy using a calibrated ^137^Cs γ-ray source (BioBeam 8000, STS, Braunschweig, Germany). All media were changed every 3 days.

### Antibodies and reagents

Antibody against MMP-2 was obtained from Santa Cruz Biotechnology, Inc. (Santa Cruz, CA, USA). HIF-1α antibody was purchased from Abcam (London, UK). Anti-β-actin antibody was purchased from Sigma-Aldrich (St. Louis, MO, USA) and was incubated with specific horseradish peroxidase-conjugated secondary antibodies (Invitrogen, Carlsbad, CA, USA). Sorafenib was purchased from LC Laboratories (Woburn, MA, USA) and solubilized in DMSO. DMSO was used in all experiments as a vehicle control. Mammospheres were cultured in serum-free MammoCult medium (Stem Cell Technologies, Vancouver, BC, Canada) containing 0.9% methylcellulose to prevent cell aggregation. Cells were harvested at various time points, fixed in 70% ethanol, and stained with 40 μg/ml propidium iodide (PI) in the presence of 50 μg/ml RNase A. Cell viability was determined using Thiazolyl Blue tetrazolium bromide (Sigma-Aldrich).

### Cell viability assay

MTT assay was conducted using a Thiazolyl Blue tetrazolium bromide and was based on the conversion of MTT to MTT-formazan by mitochondria. MCF-7 and MDA-MB-231 cells were resuspended and plated in 96-well plates at 1×10^4^ cells/200 μl in culture media with 5% FBS. Cells were incubated with or without drugs for 24–72 h then incubated with MTT (5 mg/ml in phosphate-buffered saline; PBS) for 3 h. The plate was then centrifuged at 2,000 rpm for 5 min at 4°C, and the MTT solution was removed from the wells by aspiration. The formazan crystals were dissolved in 2 ml of DMSO. The absorbance was recorded on a Paradigm Detection spectrophotometer (Beckman Coulter, Inc., Fullerton, CA, USA) at a wavelength of 540 nm.

### Cell proliferation and apoptosis analysis

Live cell numbers were counted with the ADAM-MC automated cell counter (Digital Bio, NanoEnTek Inc., Seoul, Korea). After a 72-h treatment, the cell counter measured total cell numbers, dead cell numbers, and cell viability using two sensitive fluorescence dye staining solutions, AccuStain Solution T (PI/lysis solution) and AccuStain Solution N (PI/PBS). AccuStain Solution T permeabilizes the plasma membrane and stains the nucleus, which allows total cell enumeration measurements, whereas AccuStain Solution N exclusively stains non-viable cells. A 532-nm optic laser was automatically focused on the cell suspension contained in a disposable microchip, and the cell analysis was conducted with a CDD camera. The Annexin V/PE Apoptosis Detection kit (BD Biosciences, Bedford, MA, USA) was used to assess Annexin V-positive cells. Briefly, fresh cell preparations were incubated with 1X Annexin binding buffer and Annexin V/PE (2.5 μg/ml)-conjugated primary antibody and 7-AAD (5 μl) for 15 min on ice. Following incubation, PI (10 μg/ml) was added to the suspension, and the cells were analyzed by FACSAria (BD Biosciences, San Jose, CA, USA).

### Cell cycle analysis

Cells were treated with 5 μg/ml sorafenib or 10 Gy radiation for 24 h and harvested. The cells were trypsinized and resuspended in 3 ml PBS. Cells were centrifuged and washed in 3 ml PBS. Following fixation in 70% ethanol for 16 h at −20°C, the cells were stained with PI (40 μg/ml) and RNAse A (50 μg/ml) prior to analysis. The stained cells were subjected to cell cycle analysis using FACSAria.

### FACS sorting and analysis

Cells (1×10^6^ cells) were incubated with anti-CD44-APC and anti-CD24-FITC antibodies (BD Biosciences) at 2 μg/ml (1:10) in the dark, on ice, for 15 min (MDA-MB-231) or 1 h (MCF-7), washed twice with cold PBS, then resuspended in PBS (0.5 ml). CD44^+^CD24^−^ and CD44^−^CD24^+^ cells were identified and isolated from MDA-MB-231 and MCF-7 cells using a FACSAria instrument.

### Mammosphere formation

Cells were suspended at MCF-7 (5,000 cells/ml) and MDA-MB-231 (600 cells/ml) in primary mammosphere formation and MCF-7 (1,000 cells/ml) and MDA-MB-231 (300 cells/ml) in secondary mammosphere formation in complete Mammocult media (Stem Cell Technologies) and plated in triplicate wells on a 24-well ultra-low attachment culture plate (Corning Inc., Corning, NY, USA). Cells were incubated at 37°C for 7–10 days. The number of mammospheres was imaged by inverted microscopy (Nikon Eclipse TS 100, Tokyo, Japan) and mammosphere diameters were determined using Image-Pro Plus 7.0 software. The primary mammosphere formation culture was exposed to sorafenib (5 μg/ml) and radiation (10 Gy), whereas the second passage was cultured in the absence of sorafenib and radiation. Mammospheres were collected at the second passage by gentle centrifugation (800 rpm) and dissociated enzymatically (10 min in 0.05% trypsin, 0.53 mM EDTA) and mechanically. The dissociated cells were plated in a 24-well ultra-low attachment plate and cultured for 7–10 days.

### Immunocytochemistry

Collected cell fractions from FACS were counted and cytospun onto glass slides at 1,000 cells per spot with a Cytospin 4 cytospinner (Thermo Scientific, Waltham, MA, USA). Cells were fixed in 4% paraformaldehyde and stained overnight at 4°C with primary antibodies directed against HIF-1α (1:500) followed by secondary antibodies (1:1000 Texas Red conjugated anti-mouse and anti-rabbit H+L IgG; Vector Laboratories, Burlingame, CA, USA) for 1 h at room temperature. Nuclei were counterstained with 4′,6-diamidino-2-phenylindole, and images were captured with a fluorescent microscope (Nikon Eclipse 80i).

### Western blotting

Cells were harvested with ice-cold PBS, and re-suspended in lysis buffer containing (mM) 20 Tris-HCl (pH 7.5), 150 NaCl, 1 Na_2_EDTA, 1 EGTA, 1% Triton, 2,5 sodium pyrophosphate, 1 β-glycerophosphate, 1 Na_3_VO_4_, 1 μg/ml leupeptin and 1 mM phenylmethanesulfonyl fluoride. Extracts were diluted in a mix of LDS sample buffer and heated at 95°C for 5 min. Samples were electrophoresed on 10% sodium dodecyl sulfate-polyacrylamide gels (Invitrogen) and transferred onto nitrocellulose membranes (GE Healthcare Life Sciences, Piscataway, NJ, USA). The blots were saturated in TBS-T buffer (20 mM Tris, 137 mM NaCl, 0.05% Tween-20, pH 7.6), containing 3% BSA for 1 h at room temperature, incubated overnight at 4°C with primary antibodies: anti-VEGF (1:500; Santa Cruz Biotechnology Inc.), anti-HIF-1α (1:500; Abcam), anti-β-actin (1:5,000; Sigma-Aldrich) and subsequently incubated with specific horseradish peroxidase-conjugated secondary antibodies. The immunoreactive proteins were detected using enhanced chemiluminescence (Thermo Scientific, Rockford, IL, USA). Immunoblots were quantified using the ImageMaster densitometry program.

### Statistical analysis

The paired Student’s t-test and Microsoft Excel were used for the MTT assays, cell proliferation, mammosphere formation, and FACS data, which were conducted in triplicate and repeated three times. Percent inhibition of the western blot data was determined from the ratio of band density. p-value <0.05 was considered to indicate a statistically significant difference.

## Results

### Sorafenib and radiation inhibit proliferation and induce breast cancer cell apoptosis

**Figure 1:**
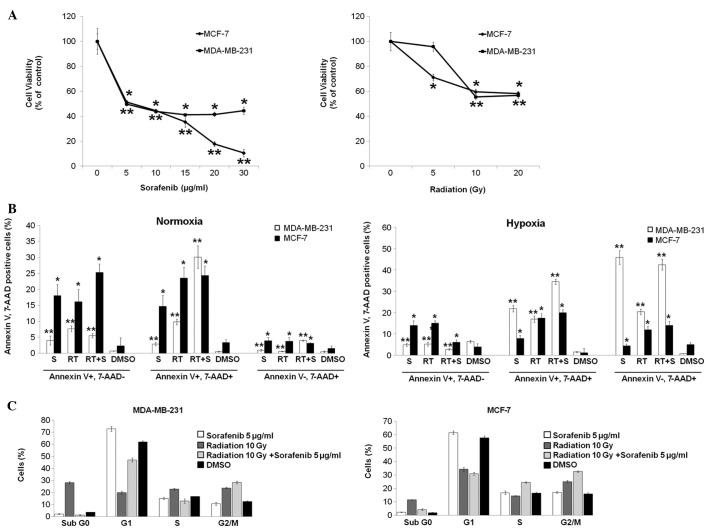
Combination of radiation and sorafenib induces synergistic apoptosis and G2/M cell cycle arrest in breast cancer cells. (A) MDA-MB-231 and MCF-7 cells (1×10^4^ cells/well) were seeded in 96-well microplates and treated with increasing doses of radiation and concentrations of sorafenib as indicated. After 72 h (radiation) and 48 h (sorafenib) of incubation, respectively, cell viability was assessed by the MTT assay, and the concentration that induced 50% growth inhibition (IC_50_) was determined to be of ~10 Gy radiation and 5 μg/ml sorafenib for both cell lines. (B) Cells were treated with radiation (RT, 10 Gy), sorafenib (S, 5 μg/ml), radiation + sorafenib (RT + S, 10 Gy + 5 μg/ml) or DMSO. After 72 h, the percentage of Annexin V/PE-positive cells was quantified using flow assisted cell sorting. (C) Cells were treated with radiation (RT, 10 Gy), sorafenib (S, 5 μg/ml), radiation + sorafenib (RT + S, 10 Gy + 5 μg/ml) or DMSO for 24 h and analyzed by flow cytometry for the cell cycle analysis. Mean percentages ± standard deviation (SD) of cells in each cycle phase are shown. Data are means of three separate experiments ^*^p<0.001, ^**^p<0.01.

To investigate the effect of sorafenib and radiation on MDA-MB-231 and MCF-7 cells, we first examined the potential to inhibit cell growth by sorafenib and radiation using MTT and apoptosis assays. Sorafenib and radiation treatment resulted in a dose-dependent inhibition of cell viability, with an IC_50_ of 5 μg/ml for sorafenib and 10 Gy of radiation in both cell lines (Fig. 1A). Furthermore, the effect of combining sorafenib and radiation was evaluated by Annexin V/PE apoptosis assay after 72 h of treatment. Cells negative for 7-AAD and positive for Annexin V were regarded as cells early in apoptosis (Annexin V+, 7-AAD−); 7-AAD-positive cells, which bind Annexin V, were defined as late apoptotic cells (Annexin V+, 7-AAD+); 7-AAD-positive cells that did not bind Annexin V were considered necrotic cells (Annexin V−, 7-AAD+). The combined treatment of sorafenib and radiation in MDA-MB-231 cells significantly increased the antitumor effect of radiation alone or sorafenib alone at 21% O_2_ (normoxia). Late apoptotic cells were observed in the combined treatment group (30.1±3.5%) compared with apoptotic cells in the sorafenib (2.9±0.5%) and radiation (9.8±0.9%) treatment groups at 1% O_2_ (hypoxia) (Fig. 1B). However, the combined treatment increased subsequent necrosis (42.5±2.5%) and it moderately increased late apoptosis (34.5±1.2%) in comparison with sorafenib or radiation treatment alone at 1% O_2_ (Fig. 1B). Hypoxia may have induced more necrosis through the effect of combined treatment in both cell lines, unlike normoxia. Similarly, early apoptotic MCF-7 cells increased under normoxia rather than under hypoxia but apoptotic cells were not clearly observed in the combined treatment group as compared with apoptotic cells in the sorafenib and radiation treatment groups under normoxia or hypoxia (Fig. 1B). These data suggest a potential synergistic effect of the combined treatment on MDA-MB-231 cells but not on MCF-7 cells. Similar results were observed in athymic nude mice bearing subcutaneously transplanted MDA-MB-231 cells (data not shown).

To determine the effect of sorafenib and radiation on cell cycle distribution, cells were synchronized by 24 h stabilization prior to the different treatments. After 24 h of treatment, G2/M arrest was observed in a flow cytometry cell cycle analysis with the combined treatment resulting in 28±1.1% (MDA-MB-231) and 32.5±0.6% (MCF-7) of the cell population compared with the control group and the single treatments (Fig. 1C).

### Combination of sorafenib and radiation inhibits breast CSCs in vitro

**Figure 2:**
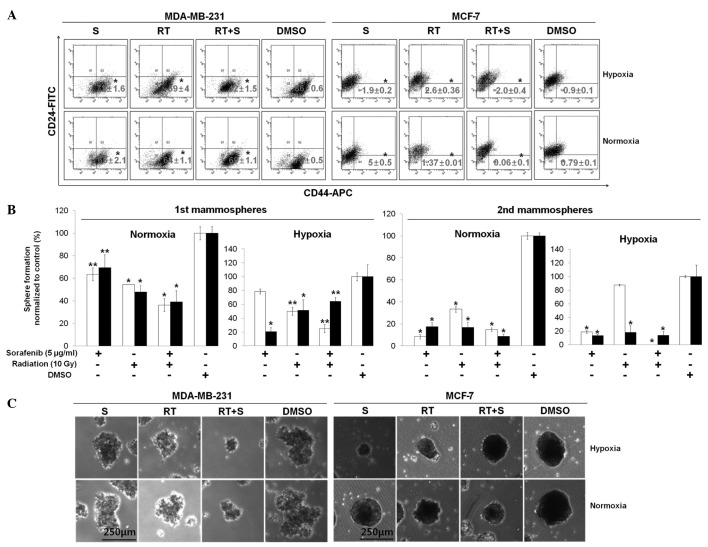
Combination of sorafenib and radiation inhibits the CD44^+^CD24^−/low^ cell population and mammosphere formation. (A) MDA-MB-231 and MCF-7 cells were treated with or without sorafenib (S, 5 μg/ml) combined with radiation (RT, 10 Gy) under 21% O_2_ (normoxia) or 1% O_2_ (hypoxia). After 72 h, a flow assisted cell sorting analysis was conducted using specific surface markers for basal (CD44-APC) and luminal (CD24-FITC) epithelial cells and the percentage of cells (CD44^+^CD24^−/low^) of each cell line was evaluated in three independent experiments. (B) Cells were seeded to form primary mammospheres at a density of MCF-7 (5,000 cells/ml) and MDA-MB-231 (600 cells/ml) and secondary mammospheres at a density of MCF-7 (1,000 cells/ml) and MDA-MB-231 (300 cells/ml). Additionally, primary mammospheres were incubated with radiation (RT, 10 Gy), sorafenib (S, 5 μg/ml), radiation + sorafenib (RT + S, 10 Gy + 5 μg/ml) or DMSO for 7–10 days, whereas secondary mammospheres were not treated with radiation or sorafenib under hypoxia or normoxia for 7–10 days. White and black bars indicate MDA-MB-231 and MCF-7 cells, respectively. Data are means of three separate experiments; bar, standard deviation (SD). ^*^p<0.001, ^**^p<0.05. (C) Representative images from primary mammospheres.

To evaluate whether the combination of sorafenib and radiation treatment could suppress breast CSCs *in vitro* under normoxic and hypoxic conditions, both cell lines were treated with 5 μg/ml of sorafenib alone, 10 Gy of radiation alone, or a combined treatment for 72 h. We assessed expression of the prospective CSC markers CD44-APC and CD24-FITC by flow cytometry. The basal cell line, MDA-MB-231 cells, showed a significantly decreased percentage of CD44^+^CD24^−/low^ cells during co-treatment with sorafenib and radiation (42±1.5%) compared to that in the sorafenib alone (72±1.6%) and radiation alone (59±4%) groups under hypoxic conditions, whereas no significant difference was observed under normoxic conditions (Fig. 2A).

Conversely, the luminal cell line MCF-7 showed a reduced percentage of CD44^+^CD24^−/low^ cells during co-treatment with sorafenib and radiation (0.06±0.1%) rather than sorafenib alone (5±0.5%) and radiation alone (1.37±0.01%) under normoxic conditions, whereas no significant difference was observed under hypoxic conditions (Fig. 2A). Thus, our data suggest that the synergistic efficacy of co-treatment with sorafenib and radiation resulted in a potential combined treatment effect for specific targeting of CD44^+^CD24^−/low^ cells from breast cancer cells *in vitro*.

A small population of breast cancer cells and mammary stem cell-like/progenitor cells are enriched in floating spherical clusters of cells (16). These properties, based on self-renewal ability, exhibit serial mammosphere formation and differentiation into multiple lineages (16). To confirm whether the combination of sorafenib and radiation could more effectively suppress mammosphere formation than single treatments such as reduction of CD44^+^CD24^−/low^ cells from co-treated breast cancer cells, cells were cultured in radiation (10 Gy), sorafenib (5 μg/ml), radiation + sorafenib (10 Gy + 5 μg/ml) or DMSO in ultra-low attachment plates and then cultured to the secondary passage in the absence of sorafenib and radiation under normoxic and hypoxic conditions. The combination of sorafenib and radiation inhibited the formation of primary mammospheres under both normoxic and hypoxic conditions (Fig. 2B), whereas these treatments increased the formation of primary mammospheres in MCF-7 cells under hypoxic conditions. The percentage of secondary mammospheres formed decreased significantly in MDA-MB-231 cells under the hypoxic conditions but not under normoxia (p<0.001) (Fig. 2B). The number of mammospheres was reduced by 1.5–87.5-fold (MDA-MB-231 cells) and 1-2.1-fold (MCF-7 cells) (p<0.05) but also the size of both primary (Fig. 2B and C) and secondary mammospheres (data not shown) decreased compared to those in single treatments. These results demonstrate that the combined treatment of sorafenib and radiation was able to preferentially target breast CSCs.

### Combination of sorafenib and radiation inhibits HIF-1α expression associated with highly activated CD44^+^CD24^−/low^ cells

**Figure 3:**
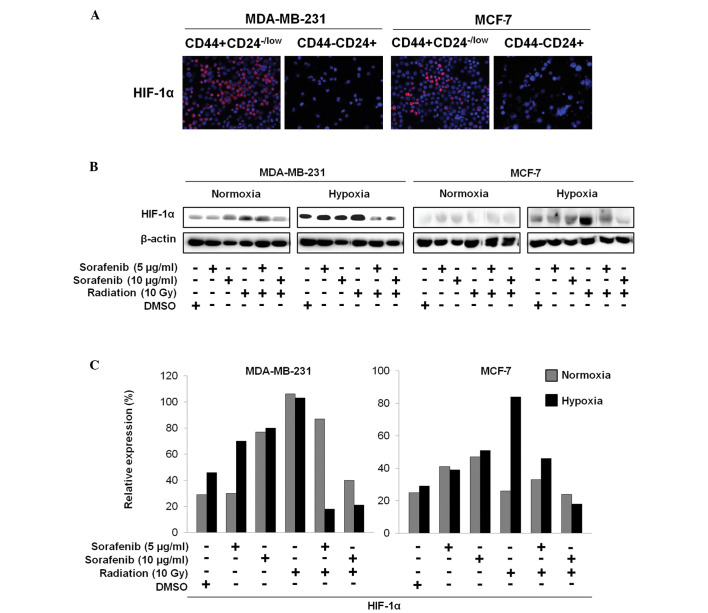
Hypoxia inducible factor (HIF)-1α from CD44^+^CD24^−/low^ cells was highly induced and the combination of sorafenib and radiation blocked HIF-1α in MDA-MB-231 cells under hypoxic conditions. (A) CD44^+^CD24^−/low^ cells were sorted by flow cytometry. Then, sorted cells were cytospun, fixed, permeabilized and immunostained with antibodies to HIF-1α and vascular endothelial growth factor (VEGF). (B) Cells were treated with radiation (RT, 10 Gy), sorafenib (S, 5 μg/ml), radiation + sorafenib (RT + S, 10 Gy + 5 μg/ml) or DMSO for 72 h. Cells were lysed and protein expression was detected by western blotting. (C) Quantification of the results from western blotting shown in (B) (normalized by the β-actin value).

The sorted CD44^+^CD24^−/low^ cells and CD44^−^CD24^+^ cells were further examined by immunocytochemistry to explore whether increased HIF-1α expression in CSCs is associated with targeting of CSCs by the combination of sorafenib and radiation (Fig. 3A). Additionally, after treatment with radiation (10 Gy), sorafenib (5 and 10 μg/ml), radiation + sorafenib (10 Gy + 5 μg/ml and 10 Gy + 10 μg/ml) or DMSO for 72 h, immunoblotting of breast cancer cells under hypoxic and normoxic conditions showed different HIF-1α expression patterns (Fig. 3B and C). HIF-1α was aberrantly expressed in the sorted CD44^+^CD24^−/low^ cells but not in the sorted CD44^−^CD24^+^ cells (Fig. 3A). As shown in Fig. 3B and C, the combination of sorafenib and radiation appeared to significantly reduce HIF-1α expression in MDA-MB-231 cells under hypoxia but was slightly decreased in MCF-7 cells under hypoxia compared to that from sorafenib or radiation alone, whereas these effects resulted in no difference under normoxia (Fig. 3B and C). Therefore, we suggest that inhibition of HIF-1α by sorafenib and radiation may eliminate breast CSCs under hypoxic conditions.

### Combination of sorafenib and radiation effectively inhibits matrix metalloproteinase-2 (MMP-2) activity in metastatic MDA-MB-231 cells

MMP-2 is well recognized for its role in tumorigenesis. Studies have shown that inhibiting MMP-2 activity reduces the metastatic potential of malignant cells and MMP-2 downregulation leads to decreased tumor cell invasion (17–21).

**Figure 4:**
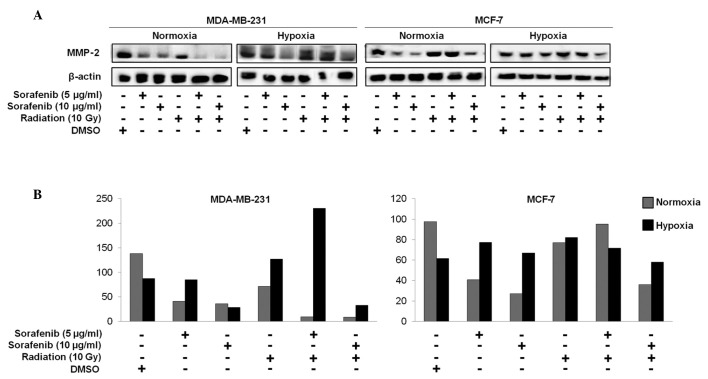
Combination of sorafenib and radiation highly reduced MMP-2 secretion from metastatic MDA-MB-231 rather than from non-metastatic MCF-7 breast cancer cells. (A) Cells were treated with radiation (RT, 10 Gy), sorafenib (S, 5 μg/ml), radiation + sorafenib (RT + S, 10 Gy + 5 μg/ml) or DMSO for 72 h. The cells were lysed and protein expression was detected by western blotting. (B) Quantification of the results from western blotting shown in (A) (normalized by the β-actin value).

We performed immunoblotting studies to investigate whether a decrease in MMP-2 could be correlated with the efficacy of combined effect of sorafenib and radiation in breast cancer. MMP-2 expression in MDA-MB-231 cells decreased in response to the combined treatment under normoxia but not under hypoxia, whereas no differences were observed in MCF-7 cells (Fig. 4A and B).

## Discussion

The anticancer efficacy of chemotherapy and radiotherapy has been evaluated in various types of cancer. However, although patients with breast cancer are treated by chemotherapy or radiotherapy, CSCs, a side population of the bulk tumor responsible for initiating and self-renewing tumor, cause relapse and metastasis and eventually give rise to new tumors (22). Therefore, we determined if the combination of sorafenib and radiation induced anti-CSC activity under hypoxia in breast cancer cells. The anticancer efficacy of sorafenib combined with radiation can be partially determined by cell cycle arrest. Sorafenib, which leads to a G1 block by inhibiting the mitogen activated protein kinase pathway, causes no obvious arrest in the majority of asynchronous cell lines, but it causes cell cycle arrest in some cell lines (23). In MDA-MB-231 and MCF-7 cells, we showed that sorafenib alone might not affect cell cycle arrest but that a combination with radiation induces G2/M arrest. Additionally, a high concentration of sorafenib is associated with inducing apoptosis via the mitochondrial intrinsic pathway and inhibits cell proliferation (24). We showed that sorafenib in combination with radiation synergistically induced apoptosis (early and late apoptosis) in MDA-MB-231 cells (5.5 and 30.1%) and MCF-7 cells (25.3 and 24.3%) compared to sorafenib alone (4 and 2.9%) and radiation alone (7.7 and 9.8%) in MDA-MB-231 cells, and sorafenib alone (18 and 14.7%) and radiation alone (16.1 and 23.5%) in MCF-7 cells under normoxia. Sorafenib may be associated with the induction of necrosis rather than apoptotic cell death, and sorafenib in combination with radiation increased late apoptosis in MDA-MB-231 cells under hypoxic conditions. Cancer cells more resistant to hypoxia show that low O_2_ tension induces apoptosis as well as necrosis and completely prevents apoptosis by Bcl-2 and Bcl-X (25). However, apoptosis and necrosis were not observed in MCF-7 cells in response to the combined sorafenib and radiation treatment under hypoxia *in vitro*.

Sorafenib has been clinically used in treatment for RCC, HCC, and thyroid cancer (12,26,27). Sorafenib alone is insufficient for inhibiting tumors in a colorectal carcinoma (HT29/*tk-luc*)-bearing animal model compared with radiation alone (28). Instead of treatment of sorafenib alone or radiation alone, a combination with sorafenib and radiation may be successfully used for treating advanced RCC (29). Similar results were also observed in metastatic MDA-MB-231 cell-bearing athymic nude mice (data not shown). Human breast cancer cells develop an increased CSC population under hypoxic conditions (7). Hypoxia-induced HIF-1α reduces migration potential and sphere formation in glioma cells and expansion of CD133^+^ CSCs in glioblastoma (30,31).

Sorafenib eliminates EZH-induced BTIC expansion, decreases the number of CD44^+^CD24^−/low^ cells, and blocks the formation of precancerous mammospheres in human breast cancer cells (13). These observations are consistent with our result that a combination of sorafenib and radiation produced synergistic inhibition of CD44^+^CD24^−/low^ cells in basal breast cancer cells (MDA-MB-231 cells) under hypoxic conditions. Furthermore, we demonstrated that the combination of sorafenib and radiation significantly suppressed mammosphere formation in MDA-MB-231 cells but not in MCF-7 cells under both hypoxic and normoxic conditions. The mechanism of suppressing breast CSCs by the combination of sorafenib and radiation is unknown, however, our results suggest that the combination of sorafenib and radiation is not cytotoxic to non-CSCs, and preferentially cytotoxic to CSCs. Accumulating evidence indicates that HIF-1α is significantly correlated with the rate of CSCs that express CD44^+^CD24^−/low^ in the early stage of breast cancer (32). Thus, HIF-1α may be a strong candidate for breast CSC-targeting with the combination of sorafenib and radiation.

This is the first *in vitro* study to demonstrate the efficacy of a combination of sorafenib and radiation for treating breast cancer cells and CSCs. The combination of sorafenib and radiation is a potentially novel strategy to inhibit breast CSCs by reducing HIF-1α and MMP-2 expression. However, these findings remain to be demonstrated in preclinical and clinical evaluations for breast cancer therapy. Nevertheless, our results clearly show for the first time that the combination of sorafenib and radiation is potentially efficacious for inhibiting breast cancer stem cells *in vitro*.
